# Malaria in infants below six months of age: retrospective surveillance of hospital admission records in Blantyre, Malawi

**DOI:** 10.1186/1475-2875-8-310

**Published:** 2009-12-29

**Authors:** Beatriz Larru, Elizabeth Molyneux, Feiko O ter Kuile, Terrie Taylor, Malcolm Molyneux, Dianne J Terlouw

**Affiliations:** 1 Liverpool School of Tropical Medicine, University of Liverpool, Pembroke Place L3 5QA Liverpool, UK; 2 Laboratorio de Inmuno-Biología Molecular. Hospital General Universitario "Gregorio Marañón", 46 Doctor Esquerdo, 28007 Madrid, Spain; 3 Department of Paediatrics, College of Medicine, University of Malawi, Box 360 Blantyre 3, Malawi; 4 Department of Infectious Diseases, Tropical Medicine & Aids, Academic Medical Center, University of Amsterdam, Faculteit der Geneeskunde, Meibergdreef 9, 1105 AZ Amsterdam, the Netherlands; 5 Department of Internal Medicine, College of Osteophatic Medicine, Michigan State University, B305 West Fee Hall, East Lansing MI 48824 Michigan, USA; 6 Malawi-Liverpool Wellcome Trust Clinical Research Programme, College of Medicine, University of Malawi, Box 360 Blantyre 3, Malawi

## Abstract

**Background:**

Information on the burden of malaria in early infancy is scarce. Young infants are relatively protected against clinical malaria during the first six months of life due to the presence of maternal antibodies and foetal haemoglobin, and have received relatively little attention with respect to research and treatment guidelines. The World Health Organization provides treatment guidelines for children from six months onwards, without specific treatment guidelines for the younger infants. A number of recent reports however suggest that the burden in this young age group may be underestimated.

**Methods:**

A retrospective review of paediatric hospital records at the Queen Elizabeth Central Hospital in Blantyre from 1998 to 2008 from three data sources was carried out. The number of admitted infants <6 months and ≤ 15 years was obtained from the registry books of the Paediatric-Nursery-Department and the Malaria Research Laboratory. For the period 2001 - 2004, more detailed malaria related admission information was available as part of an ongoing study on severe malaria, allowing a calculation of the proportion of infants < 6 months of age among admissions in children < 5 years.

**Results:**

Retrospective analysis of hospital records showed that over the course of these years, the average annual proportion of paediatric admissions in children ≤ 15 years with confirmed malaria aged <6 months was 4.8% and ranged between 2.8%-6.7%. This proportion was stable throughout the seasons. Between 2001-2004, 9.9% of admissions with confirmed malaria in children <5 years occurred in infants <6 months, with numbers increasing steadily during the first six months of life.

**Conclusions:**

These findings are consistent with recent reports suggesting that the burden of malaria during the six first months of life may be substantial, and highlight that more research is needed on dose-optimization, safety and efficacy of anti-malarials that are currently used off-label in this vulnerable patient group.

## Background

Infants below the age of six months are thought to be relatively protected against clinical malaria due to the transfer of maternal antibodies and the presence of foetal haemoglobin, and consequently receive relatively little attention with respect to research and policy guidelines on treatment of uncomplicated malaria[1,2].

However, several recent reports of facility- and community-based settings across sub-Saharan Africa include scattered information indicating a higher burden of disease in this age group than what is generally assumed and the period of protection might be shorter than the widely quoted six months [2,3]. Successful protection from malaria with insecticide-treated nets and intermittent preventive therapy targeting infants specifically (IPTi) have been associated with a marked reduction in malaria morbidity in children <6 months. Similarly, the protective effect that innate host-genetic factors, such as sickle cell trait, has throughout childhood has been recognized during the first six months of life [4,5].

Generally, reports that present data on young children do not focus on young infants specifically. For example, Bassat et al observed that nearly 8% of all paediatric malaria admissions during the years 2003-2005 occurred in infants below six months of age[6]. Eliades *et al *conducted a community survey in under 5s in Togo in 2004 and showed that the prevalence of parasitaemia increased rapidly from two months of age and the prevalence of parasitaemia with document fever remained stable from three months onwards [7]. Neither groups specifically highlight their findings in young infants.

With the current implementation of ACT across malaria control programmes in sub-Saharan Africa [8,9], the lack of attention to case-management in this group needs to be addressed.

Currently, infants <6 months are generally excluded from the regulatory trials of anti-malarials during the drug development phase. The World Health Organization provides treatment guidelines for children with weights above 5 kg and generally over five months, without specific treatment guidelines for the younger infants[10]. Clinicians working in the tropics will stress that malaria is not a rare event in the first six months of age and available first and second line oral anti-malarials are widely used off-label, based on the recommended mg/kg doses for older children. Because of the rapid changes in body composition and maturation of organs, anti-malarials likely have different pharmacokinetic profiles in the first few months of life compared to older children, potentially affecting the required therapeutic dose[11,12].

A better understanding of the burden of malaria in early infancy would help drug developers and policy makers target limited resources for research and drug development to include this group. To add to the limited data available, a study to determine the relative frequency of hospital admissions due to malaria in infants <6 months compared to older children was carried out retrospectively reviewing hospital records from the main academic hospital in Blantyre, Malawi, over an eight-year period to evaluate the need for prospective burden assessment.

## Methods

### Study area

This retrospective review was conducted in the Queen Elizabeth Central Hospital (QECH), Blantyre, Malawi, an academic hospital serving a catchment population of approximately one million. The hospital has 150 paediatrics beds, although occupation can exceed 300 at any given time. Infants ≤ 6 months with malaria are admitted to a dedicated ward for this age group: the Paediatric-Nursery-Department. The children's Accident and Emergency unit manages about 90,000 patients ≤ 15 years per year, of whom about 30% are admitted to the wards. The population catchment varies from urban in Blantyre, to semi-urban in the greater Blantyre district. Malaria transmission is seasonal with peak transmission occurring from December to June. The incidence of clinical malaria has been reported as 1.4, 0.59, and 0.11 episodes per-year among children less than five years of age, 5-15 y year old children, and adults.

### Data sources and study population

A review of the hospital records from 1998 to 2008 was carried out. Three main sources of information were available, in the years 1998, and 2005 to 2008, Information on the number of infants < 6 months of age admitted to the QECH was obtained from the registry books of the Paediatric-Nursery-Department. The information included date of admission, age in months (or in weeks or days in neonates), sex, laboratory test results including malaria smears (expressed quantitatively as negative, 1+, 2+ or 3+) and packed cell volume (PCV)]. Registry books did not record the clinical course or outcome of patients.

Information on the total number of children ≤ 15 years old admitted to the hospital with malaria (defined as a positive malaria smear on admission) was obtained from record books kept by a dedicated "Malaria Research Laboratory". This Laboratory conducts all the malaria tests for admissions to the general and research paediatric wards in children ≤ 15 years of age. The records books contain limited information, including patient identifiers, gender and lab results (including malaria smears and PCV) but not age.

For the period 2001 - 2004, more detailed information was available for individual admissions from the malaria research laboratory database and clinical records. This additional data was captured as part of an ongoing study on Severe Malaria in African Children (SMAC) as described in more detail previously[13]. The methodology to capture the absolute numbers of children admitted with malaria was essentially the same as in 1998 and 2005-2008 allowing the pooling of the datasets. The SMAC database also provided more detailed information on age, gender, and weight.

### Data management and analysis

All the information was copied from the registry and laboratory records into an Excel database. The descriptive statistical analyses were done with Excel 2007 (Microsoft Corporation, USA) and SPSS version 15 (SPSS Inc, Chicago, USA). In all analyses a malaria case was defined as any child admitted to the hospital with a positive malaria slide (asexual stages) regardless of the parasite density or disease severity.

## Results

**Figure 1:**
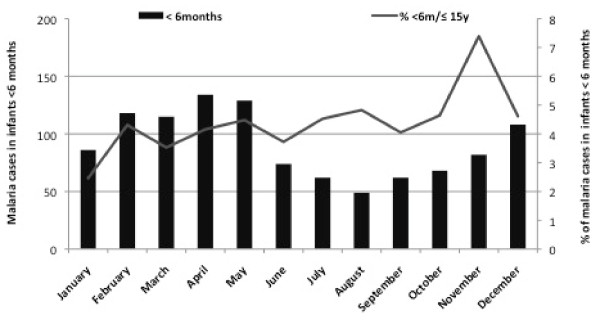
**Age distribution of malaria cases in children <15 years during the study period**. Percentages and absolute numbers of malaria cases among infants <6 months by year and by season are also shown. Total number of malaria cases per year among infants <6 months old with percentage over the 8-year period

A total of 26,413 children ≤ 15 years of age were admitted with malaria in the eight-year period (1998, and 2001 to 2007). Of these, 1,572 were admitted in the Paediatric-Nursery-Department, including 1,087 children <6 months and 439 infants aged ≤ 6 months (in 46 cases, age was not recorded) (Figure 1).

**Figure 2:**
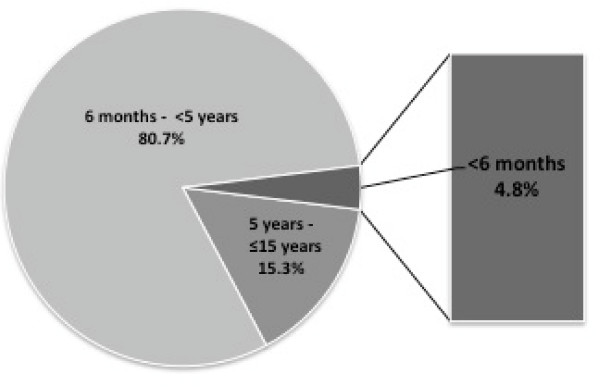
Age distribution of malaria cases in children <15 years during the study period*. The percentage of children <5 years was extrapolated from the data available during 2001-2004

The age distribution among these 1,087 infants <6 months is represented in Figure 2. The median (range) age was 4.0 months (0.1-5.0) and 49.7% of infants were male. The mean PCV value during the study period was 24.7% (SD: 8.7).

**Figure 3:**
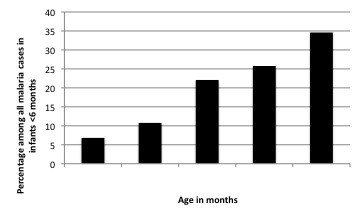
**Age distribution of malaria cases in infants <6 months during the study period**.

**Figure 4:**
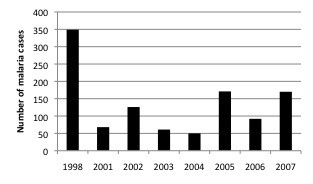
**Total number of malaria cases among infants <6 months during the study period**.

The average annual number of admissions with positive malaria parasitaemia in all age groups was 3,302 (SD: 2831), with a peak of admissions in 1998 (8947 patients), with 25,326 children <15 yr and 1,087 infants < 6 months (Figure 3). The average of the annual proportion of malaria cases aged <6 months among the paediatric admissions with positive malaria parasitaemia over the course of these years, was 4.8% and ranged between 2.8%-6.7% (Figure 4). While there was a strong seasonal trend in malaria transmission and admission numbers, the proportion of paediatric malaria admissions aged <6 month was relatively stable throughout the year (Figure 4).

### SMAC data: 2001-2004

**Figure 5:**
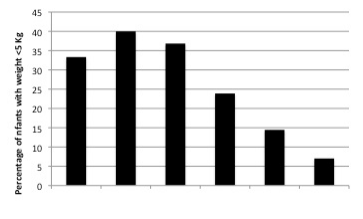
**Percentage of infants with weight <5 Kg among all malaria cases in infants <6 months during 2001-2004 according to age**.

A more detailed analysis by age group could be conducted for the years 2001-2004. During this period, 83.7% of the paediatric malaria admissions were aged <5 years old, 46.9% <2 years, 23.1% <12 months, 8.3% <6 months and 0.5% <2 months old. In other words, 9.9% of malaria admissions in the under-5s were infants below 6 months. The average weight of 305 infants <6 months of age admitted with malaria was 6.2 kg (SD: 0.3) during the 2001-2004 when body weight data was collected, Among this group, 47 infants (15.4%) had a weight <5 kg and 63.3% weighted between 5-7.5 Kg. The minimum weight registered during the study period was 2.3 Kg (Figure 5).

## Discussion

Between 1998 and 2007, on average 4.8% of children (≤ 15 yrs) admitted to the Queen Elizabeth Central Hospital in Blantyre with confirmed malaria parasitaemia per year were aged <6 months. This proportion was broadly similar across the seasons and years.

This finding supports a growing number of reports suggesting that clinical malaria in infants <6 months old is not a rare event. Although the risk is less than in older infants, it appears to increase steadily from early infancy onwards[14]. Eliades *et al *in Togo also observed a rapidly increasing parasite prevalence and clinical malaria in a random sample of children at community-level from the age of two months onwards; with parasitaemia prevalence increasing from 18.2% in children aged 0-2 months to 43.0% in children aged 3-5 months, and the prevalence of parasitaemia with document fever remained stable from three months onwards [7]. Afolabi *et al *reported similar findings in a study conducted in Nigeria, where 27.1% of all the admitted infants ≤ 6 months had positive malaria parasitaemia, and malaria rates increased after the second month of life[3]. Bloland *et al *estimated that 59.0% of febrile episodes during the first five months of life in western Kenya were attributable to malaria[15]. Alonso and colleagues in Mozambique reported that children <6 months accounted for more than 10% of all the registered paediatric out-patient visits (almost 10,000 over a period of 2 years and admitted infants <6 months with malaria represent 4% of the malaria related admissions in children <15 years[6,16].

While our findings contribute to the available data, this retrospective review has several clear limitations. Because of its retrospective nature and reliance on mostly routine hospital records the quality and completeness of the available data varied over the years. The main finding of this study is the annual proportion of young infants admitted (4.8%), but this may underestimate the absolute number of malaria cases in all age groups that were admitted. Not all cases were recorded in the Registration Book at the time of admission, and malaria smears may not have been done for all children at the time of admission due to time and staff constraints in a poor-resource setting. Neonates with congenital malaria were not included either, as they were admitted to the neonatal ward. A second limitation is that multi-source data may generate some (biased) level of error when all the data are pooled together. Furthermore, these in-patient data are not necessarily representative of the age distribution of clinical malaria in children attending the out-patient clinics, or in the population. The greater amount of malaria cases detected in 1998 can be explained due to the effect that the meteorological phenomenon of *El Niňo *had on the level of rainfall and the subsequent increase in malaria transmission during this particular year. Despite this considerable increase in malaria transmission data form 1998 was poled with the rest of the years included in the study because the proportion of infants under six months admitted with malaria during 1998 (3.9%) was similar to the one reported among the rest of the study period. A similar season distribution in malaria cases among infants was observed in 1998 compared with the rest of the study period.

Despite these limitations, the results of this retrospective review are consistent with a growing number of observations from both facility and community-level assessments suggesting a higher burden in early infancy than generally assumed. Children below five kg are not included in the WHO treatment guidelines for the new ACT that are currently being implemented across the globe. Infants under six months of age have also been systematically excluded from the efficacy and safety trials that have been conducted as part of the standard drug development process for ACT[17]. Despite the lack of evidence-based guidelines, and mainly due to the absence of alternative recommended treatment options, clinicians across Africa do use these anti-malarials on a daily basis in this vulnerable age group, using pragmatic experience to guide their decisions on dosage. With the widely accepted notion that pharmaco-kinetics and pharmaco-dynamics profile change considerably in the first few months of life, it is crucial that the current effort towards programmatic implementation of ACT needs to include this group.

## Conclusion

The burden of malaria during the six first months of life is probably underestimated, and may represent up to 5% of all admitted paediatric malaria cases in children below 15 years. With the wide-scale implementation of artemisinin-based combination therapy (ACT) for the case-management of uncomplicated malaria throughout sub-Saharan Africa, accurate estimates of burden, pharmacokinetic profiles, safety and efficacy of ACT in early infancy are urgently needed to guide the development of evidence-based treatment guidelines for the different artemisinin-based combinations that are currently used off-label in this vulnerable patient group.

## Competing interests

The authors declare that they have no competing interests.

## Authors' contributions

BL, EM and DJT developed the surveillance methods. EM, MM and TT contributed data and supported the compilation of data conducted by BL. BL conducted the analyses, with support from DJT, and prepared the initial draft. All authors contributed to the development of the final manuscript.
